# Water hammer in pipelines based on different friction models

**DOI:** 10.1038/s41598-024-51409-9

**Published:** 2024-01-10

**Authors:** Dan Jiang, Chen Zeng, Qixia Lu, Qing Guo

**Affiliations:** 1 School of Mechanical and Electrical Engineering, University of Electronic Science and Technology of China, Chengdu, China; 2 School of Aeronautics and Astronautics, University of Electronic Science and Technology of China, Chengdu, China

**Keywords:** Fluid dynamics, Mechanical engineering

## Abstract

Water hammer in pipelines is a difficult problem in fluid transmission field. Especially, there exists some friction items of pipeline transient model such that the simulation model is not consistent to the experimental results. By using the friction model proposed by Kagawa and the model of impulse response function, the pressure transients are calculated with and without cavitation. The corresponding simulation results involving pressure, velocity, steady and dynamic frictions, cavitation volume are analyzed to reveal the effect of friction item on pressure transients. Moreover, the features of steady and dynamic frictions are captured in pipelines with upstream and downstream valves. The comparative simulation results of two friction models have verified that the friction model using an impulse response function has higher consistency between simulation and experimental results of pipeline transients.

## Introduction

Water hammer phenomenon usually appears in pipelines due to instant decelerated flow by rapid valve closure, and even leads to hydraulic pipeline crack. However, during pipeline transients, it has not standard model of friction items in previous studies.

For laminar flow, Zielke^1^ derived an additional friction item related to the dynamic friction equation during transients, where involves both fluid accelerations and weighting functions. However, the numerical algorithm requires a large amount of computer memory and computation time. To improve the computation efficiency, many researchers^2–4^ investigated the friction model in transient laminar flow. Trikha presented three estimated weighting functions to construct the dynamic friction. Then Kagawa et al.^5^ and Taylor et al.^6^ respectively proposed ten and four estimated weighting functions to improve the calculation accuracy of the friction model. Moreover, Vardy and Brown^7,8^ developed the friction model into turbulent smooth and fully rough pipeline flows. Brunone et al.^9–11^ proposed the instantaneous acceleration-based (IAB) model and investigated the effect of initial Reynolds number on dynamic friction model. Szymkiewicz and Mitosek^12^ presented alternative convolution approach to address the friction item in unsteady pipelines. An analytical expression of weighting functions^13–16^ during water hammer was proposed. However, the effectiveness of friction models needs to be further verified in cavitation conditions.

In low pressure hydraulic pipelines, the pressure variation induces cavities growth and collapse, which is also called vaporous cavitation. Generally, the discrete gas cavity model (DVCM)^17,18^ and the discrete vapour cavity model (DGCM)^19–21^ are two classic cavitation models. Then Zhou et al.^22^ used a 2-order finite volume method to capture the growth and collapse of vaporous cavitation.

In order to accurately predict pressure transients in pipelines, a reasonable friction model should be constructed to guarantee the simulation consistent to the experimental results under both with and without cavitation. By using the impulse response function to construct the dynamic friction model, the corresponding simulation results involving pressure, velocity, steady and dynamic friction, cavitation volume are analyzed. The comparison results with Kagawa^5^ model have been verified to the effectiveness of the friction model using an impulse response function.

The remainder of this paper is organized as follows. The water hammer model in hydraulic pipelines is constructed in Sect. "Mathematical model of water hammer". The comparative results verification between simulation and experiment are given in Sect. "Simulation results". Finally, the conclusions are drawn in Sect. "Conclusions".

## Mathematical model of water hammer

### Basic equations of transient flow

**Figure 1:**
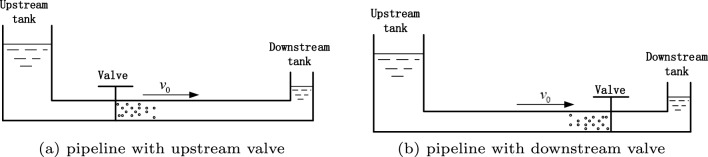
Tank-pipeline-valve system.

With valve sudden closure, water hammer is the transmission of pressure wave because of rapid change of instantaneous velocity. The tank-pipeline-valve system following a sudden shut-off valve is illustrated in Fig. 1, including pipeline with upstream valve and pipeline with downstream valve.

In pipeline with upstream valve, with valve rapid closure, the velocity at the valve decreases suddenly to zero. There is the trend of flow away from the valve, so the pressure also drops rapidly. While in pipeline with downstream valve, with valve rapid closure, the velocity reduces instantly to zero. There is the trend of flow continuing to approach the valve, so the pressure rapidly rises to the maximum valve.

This phenomenon is commonly described by the continuity equation and the momentum equation^23^. The continuity equations of gas and liquid phase are

1 $$\begin{aligned} \frac{\partial }{\partial t}(\rho _g \alpha A_p)+\frac{\partial }{\partial x}(\rho _g \alpha A_p v)=\Gamma A_p, \end{aligned}$$

2 $$\begin{aligned} \frac{\partial }{\partial t}[\rho _l (1-\alpha ) A_p]+\frac{\partial }{\partial x}[\rho _l (1-\alpha ) A_p v]=-\Gamma A_p, \end{aligned}$$

where $\rho _g$ is the density of the gas phase(kg/m$^3$), $\rho _l$ is the density of the liquid phase(kg/m$^3$), $\alpha$ is the ratio of the volume of the gas phase to the volume of the fluid, $A_p$ is piping cross-sectional area(m$^2$),and $\Gamma$ is the flow function, which is the mass exchange rate between liquid and gas phases.

Assuming $A_p$ is constant, add the Eqs. (1) and (2) to get:

3 $$\begin{aligned} \frac{\partial }{\partial t} \left\{ [\rho _g \alpha + \rho _l (1-\alpha )] \right\} +\frac{\partial }{\partial x} \left\{ [\rho _g \alpha + \rho _l (1-\alpha )]v \right\} =0. \end{aligned}$$

Meanwhile, the momentum equation is

4 $$\begin{aligned} \frac{\partial }{\partial t} \left\{ [\rho _g \alpha + \rho _l (1-\alpha )]v \right\} +\frac{\partial }{\partial x} \left\{ [\rho _g \alpha + \rho _l (1-\alpha )]v^2 \right\} + \frac{\partial p}{\partial x} + F(q)=0, \end{aligned}$$

where *p* is the pressure in the pipeline(Pa), *q* is the flow rate (m$^3$/s), *F*(*q*) is the friction term.

### Cavitation model

Cavitation can occur during water hammer. When the pressure in the pipeline drops to the vapor pressure of the liquid, vapour cavities will form, and the cavities will be collapsed as the pressure rises again.

According to the flow continuity principle, the dynamics of the cavitation volume $V_{cav}$ can be described by DVCM. The DVCM is a relatively simple model for simulating cavitation volume in transient flow^18^:

5 $$\begin{aligned} {V_{cav}}=\int {(q_{2}-q_{1})}{dt}, \end{aligned}$$

where $q_{1}$ and $q_{2}$ are the upstream flow rate and downstream flow rate of an element in the pipe, respectively.

### Friction models

In traditional mathematical model of water hammer, it is assumed that the friction with transient flow is equal to steady friction. However the traditional model of water hammer cannot effectively predict the pressure pulsation process^24^. So the friction in Eq. (4) is described as the sum of the steady friction item and the dynamic friction item:

6 $$\begin{aligned} {F(q)}={F_0}+{F_f}, \end{aligned}$$

where the first item $F_0$ is the steady friction, and the second item $F_f$ is the dynamic friction. The steady friction $F_0$ can be expressed as

7 $$\begin{aligned} F_0=\frac{{\rho _m fq\left| q \right| }}{{4\pi ^2r^5}}, \end{aligned}$$

where *f* is the Darcy-Weisbach friction factor, $\rho _m$ is the density of mixture, *r* is the radius of pipeline.

#### Kagawa model

**Figure 2:**
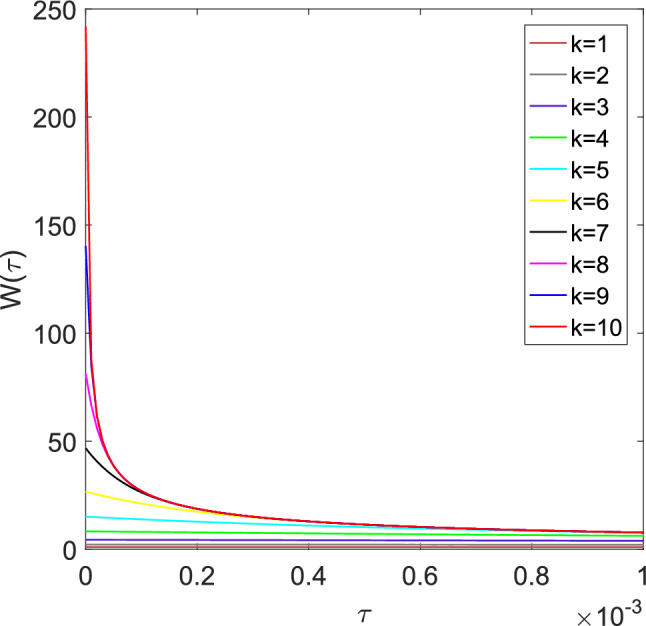
The weighting function $W(\tau )$ proposed by Kagawa.

To improve calculation efficiency, Kagawa^5^ developed a more precise procedure on the basis of three weighting terms for Trikha^2^. The weighting function can be described as the sum of *k* impulse responses of first order elements:

8 $$\begin{aligned} W(\tau ) \approx \sum _{i=1}^{k} {W_i(\tau )}=\sum _{i=1}^{k} {m_i e^{-n_i \tau }},\quad k=1,2,\cdots ,10 \end{aligned}$$

where *k* is determined according to $\Delta \tau /2>{\tau _m}_i$ ($\Delta \tau ={\mu _m \Delta t}/ {\rho _m r^2 }$)^25^, and the constants $n_i$, $m_i$ and ${\tau _m}_i$ are listed in Table 1. The curves of $W(\tau )$ are shown in Fig. 2.

So the dynamic friction of Kagawa model can be computed by

9 $$\begin{aligned} F_f=\frac{1}{2}\sum _{i=1}^{k} Y_i(t), \end{aligned}$$

10 $$\begin{aligned} Y_i(t)=\int _{0}^{t} {W_i(t-u) {\frac{\partial F_0(u)}{\partial t}}du}. \end{aligned}$$

So each $Y_i$ in the subsequent time step can be calculated as follows:

11 $$\begin{aligned} \left\{ \begin{array}{l} \\ Y_i(t+{\Delta t}) = Y_i(t)e^{-n_i{\Delta \tau }} + m_ie^{-n_i\frac{\Delta \tau }{2}} [F_0({t+{\Delta t}})\\ \qquad -F_0(t)]\\ \\ Y_i (0) = 0 \\ \end{array}. \right. \end{aligned}$$

**Table 1:** $$n_{i}$$, $$m_{i}$$ and $${\tau _m}_i$$.

i	$n_i$	$m_i$	${\tau _m}_i$
1	$2.63744 \times 10^{1}$	1.0	$6.2 \times 10^{-2}$
2	$7.28033 \times 10^{1}$	1.16725	$2.8 \times 10^{-2}$
3	$1.87424 \times 10^{2}$	2.20064	$9.9 \times 10^{-3}$
4	$5.36626 \times 10^{2}$	3.92861	$3.3 \times 10^{-3}$
5	$1.570606 \times 10^{3}$	6.78788	$1.1 \times 10^{-3}$
6	$4.61813 \times 10^{3}$	$1.16761 \times 10^{1}$	$3.6\times 10^{-4}$
7	$1.36011 \times 10^{4}$	$2.00612 \times 10^{1}$	$1.2\times 10^{-4}$
8	$4.00825 \times 10^{4}$	$3.44541 \times 10^{1}$	$4.1 \times 10^{-5}$
9	$1.18153 \times 10^{5}$	$5.91642 \times 10^{1}$	$1.4 \times 10^{-5}$
10	$3.48316 \times 10^{5}$	$1.01590 \times 10^{2}$	$4.7 \times 10^{-6}$

#### Model using an impulse response function

Different from Kagawa model using *k* terms of impulse response functions, in this paper the model is mainly used an impulse response function. This model is defined in the real time domain, which is not related to the assumed viscosity distribution over the pipe’s cross section^12^. It can be given as follows:

12 $$\begin{aligned} h(t)=\frac{1}{K}e^{(\frac{-t}{K})}. \end{aligned}$$

So the dynamic friction can be expressed by:

13 $$\begin{aligned} F_f=k_1 X(t), \end{aligned}$$

in which

14 $$\begin{aligned} X(t)=\int _{0}^{t} {h(t-u) {\frac{\partial F_0(u)}{\partial t}}du}, \end{aligned}$$

with

15 $$\begin{aligned} \left\{ \begin{array}{l} \\ X(t+{\Delta t}) = X(t)e^{-\frac{\Delta t}{K}} + \frac{1}{K}\cdot e^{-\frac{\Delta t}{2 K }} [F_0({t+{\Delta t}})\\ \qquad -F_0(t)]\\ \\ X(0) = 0 \\ \end{array}. \right. \end{aligned}$$

where $k_1$ in Eq. (13) is an empirical coefficient, $\Delta t=r^2 \Delta \tau \rho _m/{\mu _m }$ and *K* is a parameter expressed in time unit. Here $k_1$ is related to flow rate and pipeline characteristics, which determines the size of dynamic friction terms.

Here *K* determines the convergence of the impulse response, which is related to the pressure wave velocity and pipe length. Using a priority assumed flow memory $M=4T$ to calculate the value of *K*, *T* is the period of pressure wave oscillation ($T=4L/a$). The value of *K* can be calculated for each different cases based on the assumed convergence tolerance $\varepsilon$ ($\varepsilon$ is a small positive number) and flow memory *M*^26^. In Eq. (12), when *t* is equal to *M*, $\varepsilon$ is written as follows:

16 $$\begin{aligned} \varepsilon =\frac{1}{K}e^{(\frac{-M}{K})} . \end{aligned}$$

In the model using an impulse response function, *K* is an important parameter to determine the convergence of function, which can be calculated by Eq. (16). In the same pipeline(*L*=37.2 m and *r*=11.05 mm), the values of *K* are different when the pressure wave velocity is different, as listed in Table 2. The pressure wave velocity *a* can be calculated by:

17 $$\begin{aligned} a=\sqrt{\frac{K_m}{\rho _m}}, \end{aligned}$$

where $K_m$ is the bulk modulus of mixture, which ranges from 16e8 Pa to 21e8 Pa. Here $\varepsilon =0.001$ in Eq. (16). It can be noted that *K* drops from 0.048 s to 0.040 s as the pressure wave velocity increases from 1265 m/s to 1476 m/s.

**Table 2:** Values of parameter *K*.

Pressure wave velocity *a* (m/s)	*M* (s)	*K* (s)
1265	0.471	0.048
1380	0.431	0.043
1476	0.403	0.040

**Figure 3:**
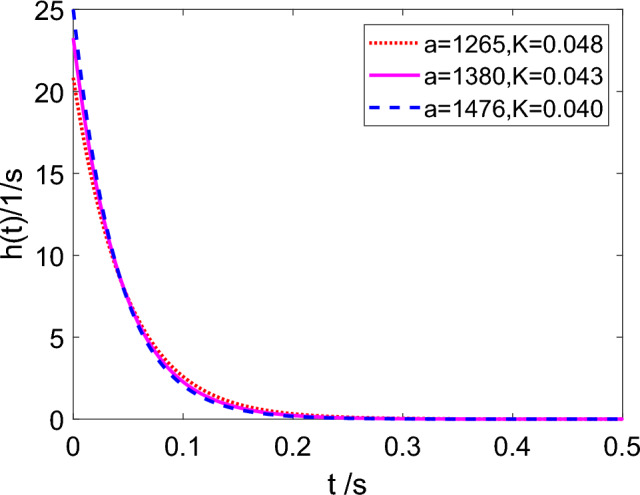
The image of impulse response function.

In order to investigate the effect of *K*, the plots of *h*(*t*) in Eq. (12) corresponding to the selected values of the parameter *K* are shown in Fig. 3. The convergence rate of impulse response function increases as *K* decreases.

## Simulation results

The aim is to verify the prediction effect of different friction models on water hammer process in hydraulic pipelines, especially for the pressure transients accompanying vaporous cavitation. The following simulation results of pressure transients without cavitation and with cavitation are based on two different friction models (Kagawa model and the model using an impulse response function).

### Pressure transients without cavitation

The experimental results of transient pressure pulsations in the pipeline with upstream valve are given by Vitkovsky et al.^27^ and the related parameters of tested pipeline are listed in Table 3.

Here, the weighting function of Kagawa model is approximated by sum of nine impulse responses of first order elements ($k=9$ in Eq. (8)). The pressure wave period of oscillation *T* is equal to 0.108 s ($T=4L/a$). So the flow memory can be calculated $M=0.431$ s. The parameter *K* in Eq. (16) is calculated as 0.043 when the convergence tolerance $\varepsilon =0.001$. Another element number $k_1$ in Eq. (13) is provided by comparing the numerical results and the experimental data, which refers to the trial and error method in Szymkiewicz and Mitosek^12^. Finally the value of parameter $k_1$ is approximately equal to 2.1, which determines the magnitude of dynamic friction force.

**Table 3:** Parameters of pressure transients without cavitation from Vitkovsky.

Parameters	Values
Upstream tank pressure $p_{resu}$ (bar)	4.25
Downstream tank pressure $p_{resd}$ (bar)	4.22
Pipe radius *r* (mm)	11.05
Pipe length *L* (m)	37.2
Water density $\rho$ ($\text {kg}/\text {m}^{\text {3}}$)	1000
Initial velocity $v_0$ (m/s)	0.3
Pressure wave velocity *a* (m/s)	1319

**Figure 4:**
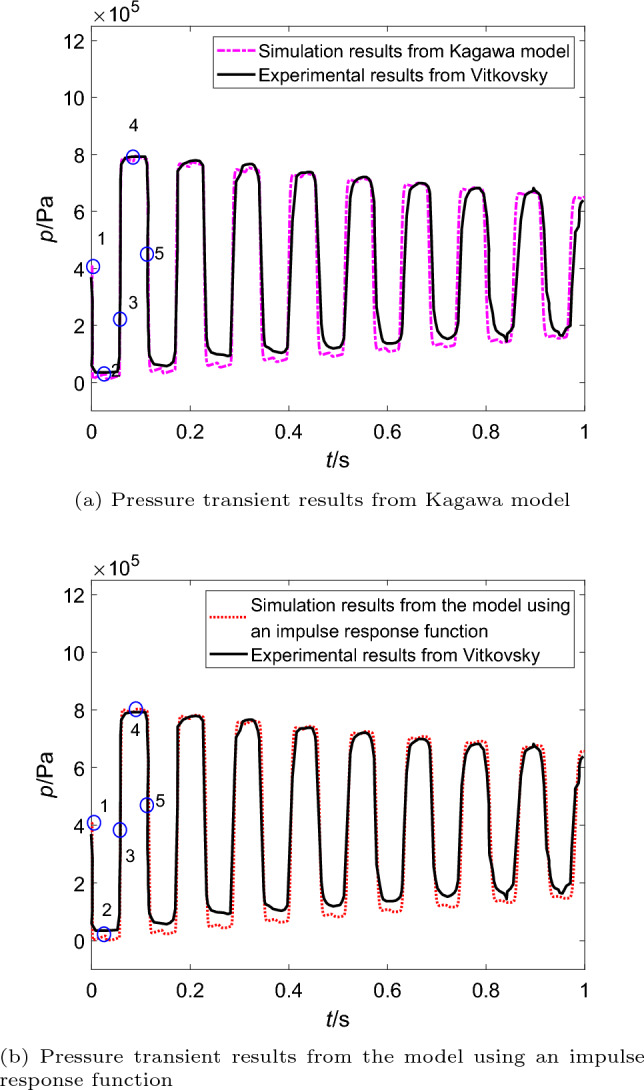
Comparison of simulation and experimental pressure transients without cavitation.

The corresponding experimental results of transient pressure pulsations close to the valve are shown as the solid line in Fig. 4. It is clear that the pressure wave gradually decays due to the friction force. And the simulation results of two different friction models are also compared (Fig. 4a from Kagawa model and Fig. 4b from the model using an impulse response function), and points 1 to 5 remarked in two figures represent a period of pressure pulsations. Here the point 1 is the valve closing time spot.

It can be observed in Fig. 4 the simulation results of the model using an impulse response function is more consistent than the simulation results of Kagawa model with the experimental pressure results. The discrepancies between the results from two models are magnified about from 0.4 to 1 s. Results from Kagawa model show evident difference compared with the experimental data (Fig. 4a), and the magnitude of discrepancies in phase shift is larger than the magnitude of discrepancies in attenuation of the pressure wave. However, the simulation results from the model using an impulse response function (Fig. 4b) are still keep the best fit, and there is no evident phenomenon of pressure pulsation leading or lagging.

**Figure 5:**
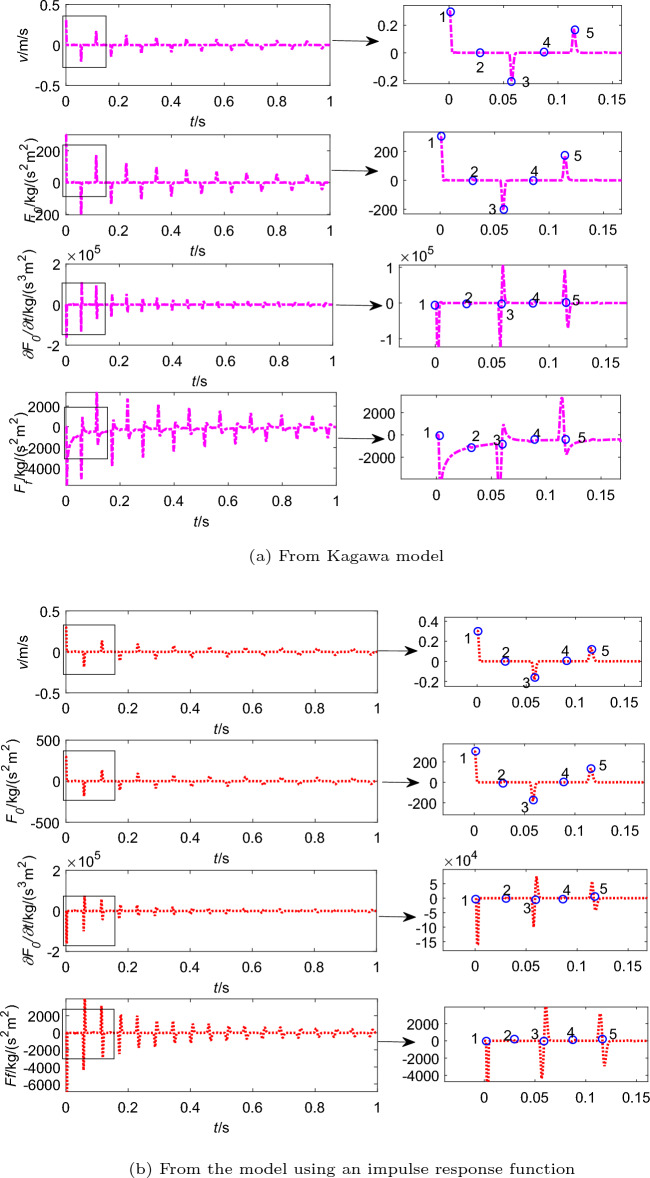
Steady and dynamic frictions without cavitation (in the pipeline with upstream valve).

In addition, Fig. 5 shows other information from two different friction models, including flow velocity (*v*), steady friction ($F_0$), the time derivative of the steady friction (${\partial F_0}/{\partial t}$) and dynamic friction ($F_f$). And the process from point 1 to 5 is a cycle, which corresponds to Fig. 4.

In pipeline with upstream valve, the flow process can suddenly be blocked when the valve is rapidly closed, which causes the flow velocity decreases rapidly to zero (point 1 to 2 in Fig. 5). At the moment, the shock pressure ($-\Delta p$) is generated because the kinetic energy is converted to the pressure energy, so the pressure drops to $p_0-\Delta p$. Afterwards, the shock pressure is equal to $\Delta p$ because the pressure energy is converted to the kinetic energy at the downstream tank, and the pressure rise to $p_0$ (point 2 to 3). In the period of point 3 to 4, the flow velocity suddenly becomes zero because the valve is fully closed, which causes pressure rise to $p_0+\Delta p$. Then the pressure wave travels downstream again and the flow velocity goes up to $v_0$ and pressure energy is converted to the kinetic energy, so the pressure back to $p_0$ (point 4 to 5). This process may be repeated several times before the fluid energy is dissipated.

And the steady friction force oscillates in phase with the flow velocity. As the flow velocity tends to zero, $F_0$ tends to zero as well. However, the dynamic friction is gradually decreases as shown in Fig. 5, which is determined by the time derivative of the steady friction, i.e., ${\partial F_0}/{\partial t}$. The extreme value of the dynamic friction is generated at the moment when the extreme value of the derivative occurs (as shown in Fig. 5a and b ). Obviously, the magnitudes of dynamic friction according to Kagawa model and the model using an impulse response function are different. Compared between Fig. 5a and b, the dynamic friction force from Kagawa model decays faster than the model using an impulse response function, which causes the pressure wave from Kagawa model slightly ahead with the experimental results.

In Fig. 4, it also can be found that the simulated pressure value are always greater than the saturated vapour pressure.Thus, there is no cavitation occurred in pipelines.

### Pressure transients with cavitation in pipeline with upstream valve

Several cycles of cavity formation and collapse occur before the minimum pressure in the upstream pipeline remain permanently above the vapour pressure. In order to test the efficiency of the model using an impulse response function for transient pressure pulsations with cavitation, the upstream pressure transient pulsations in the low pressure horizontal pipeline was also investigated. Some experimental parameters from Sanada et al.^28^ are listed in Table 4.

**Table 4:** Parameters of pressure transients with cavitation from Sanada.

Parameters	Values
Upstream tank pressure $p_{resu}$ (bar)	5.49164
Downstream tank pressure $p_{resd}$ (bar)	0.98065
Pipe radius *r* (mm)	7.6
Pipe length *L* (m)	200
Water density $\rho$ ($\text {kg}/\text {m}^{\text {3}}$)	1000
Initial velocity $v_0$ (m/s)	1.5

In this case, the weighting function of Kagawa model is approximated by sum of six impulse responses of first order elements ($k=6$ in Eq. (8)). The pressure wave period of oscillation $T=1.125$ s, so the flow memory $M=4.501$ s. When the convergence tolerance $\varepsilon$ is assumed as 0.001, the corresponding parameters in the model using an impulse response function can be calculated as follows: $K=0.611$ s and $k_1=1.91$.

**Figure 6:**
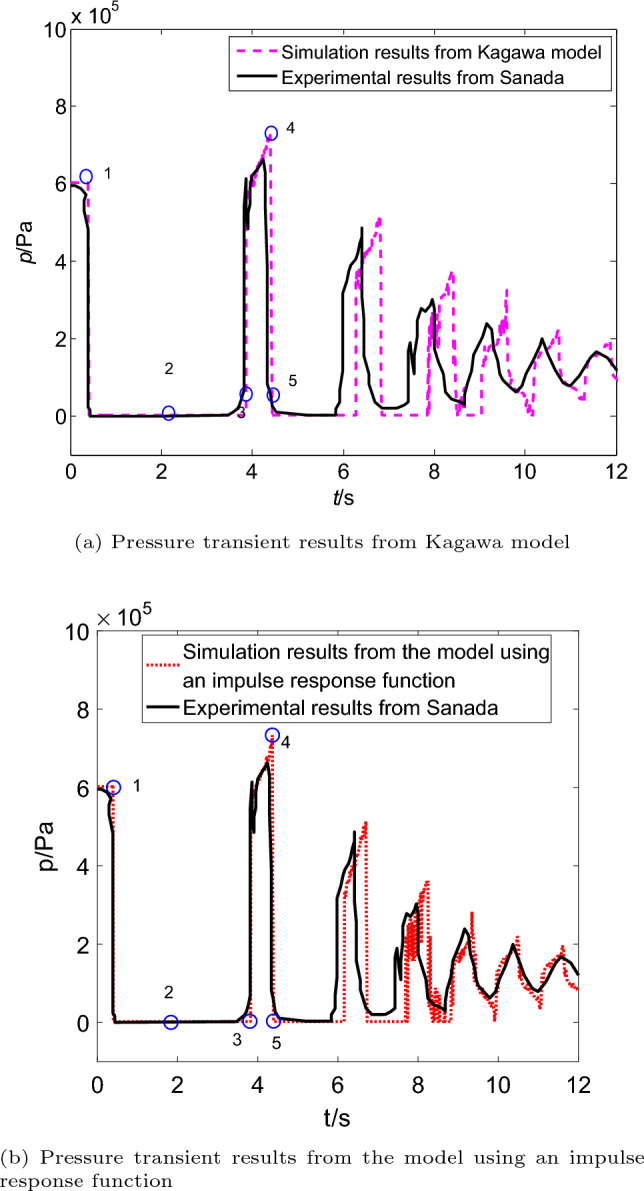
Comparison of simulation and experimental pressure transients with upstream cavitation.

The corresponding experimental results are shown as the solid line in Fig. 6. The simulation results obtained from the two different friction models (Kagawa model and the model using an impulse response function) are also reported in Fig. 6. At the beginning of pressure transients, there is a good agreement between the experimental pressure data and simulation results from Kagawa model (Figure 6a) and the model using an impulse response function (Fig. 6b). However, there are phase differences in the subsequent peaks from Kagawa model, which is that the simulated pressure lags behind the experimental results. Comparison of the results of two models demonstrates that the model using an impulse response function is also reasonable for predicting the pressure transients with cavitation in upstream pipeline, and clearly the simulation results of the model using an impulse response function provides the better accuracy.

**Figure 7:**
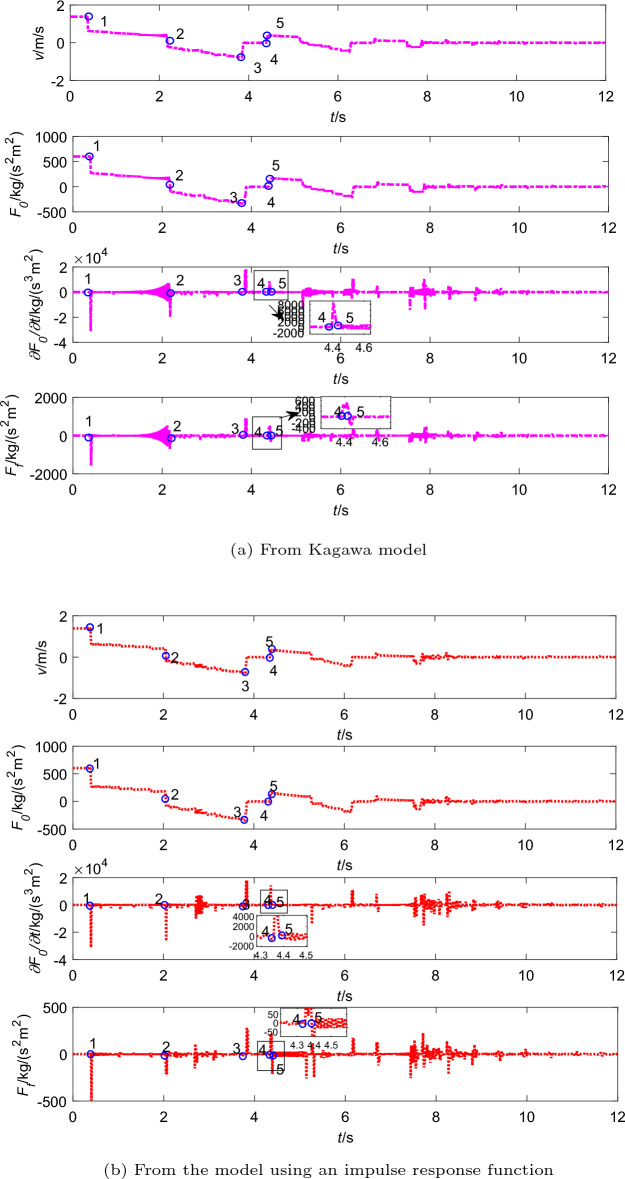
Steady and dynamic frictions with cavitation (in the pipeline with upstream valve).

In Fig. 7a and b, the flow velocity does not immediately drop to zero due to cavitation (point 1 to 2). But it is same as the case of no cavitation that the steady friction gradually decreases with flow velocity tends to zero. And its extreme value occurs before it is transient, when the flow is steady and flow velocity has the greatest value as shown in Fig. 7. The dynamic friction varies with the time derivative of the steady friction. The difference between the dynamic friction analysis with cavitation and without cavitation is that the dynamic friction has a high numerical oscillation in the period of cavitation occurring.

The cavitation volume in the element close to the valve are also predicted by Kagawa model and the model using an impulse response function. The vaporous cavitation processes obtained from the experimental pressure pulsation in Fig. 6 are listed in Table 5. The trends of cavitation change simulated by the two models are also listed, which includes the start time, the end time and the duration. It can be seen that the first cavitation collapses at 3.903 s (Kagawa model) and 3.885 s(the model using an impulse response function) from Table 5.

**Table 5:** Trends of cavitation generates and collapses in the upstream pipeline.

Times		Results from Kagawa model	Results from the model using an impulse response function
1st time	Start time (s)	0.434	0.434
	End time (s)	3.903	3.885
	Duration (s)	3.469	3.451
2nd time	Start time (s)	4.485	4.457
	End time (s)	6.322	6.210
	Duration (s)	1.837	1.753
3rd time	Start time (s)	6.894	6.782
	End time (s)	7.907	7.729
	Duration (s)	1.013	0.947

**Figure 8:**
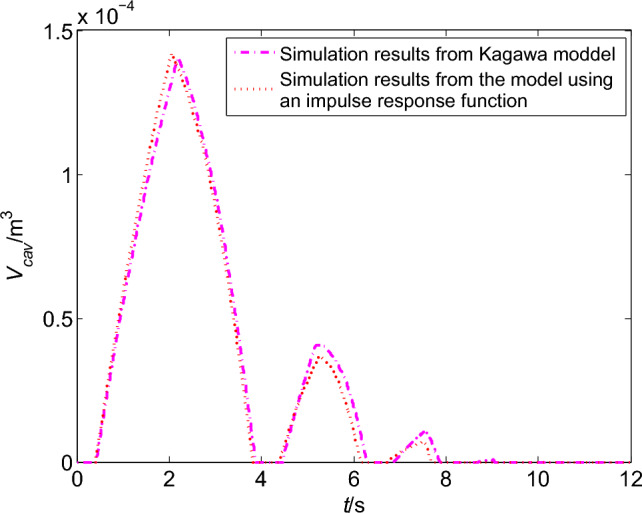
Comparison of the cavitation volume in upstream pipeline.

The corresponding cavitation volumes are shown in Fig. 8. The maximum size of the first vaporous cavity from Kagawa model is almost the same as the model using an impulse response function. When the pressure falls again, cavitation is generated again, but it is much smaller than the first cavity. Once again the cavity collapse arrival of the third pressure peak in Fig. 8. At last, the cavities experience three times of growth and collapse. And the volumes of the last two cavities from the model using an impulse response function are slightly smaller than Kagawa model.

### Pressure transients with cavitation in pipeline with downstream valve

As same as upstream pipeline, cavitation also occurs in the downstream pipeline when the pressure at the vicinity of the valve is lower than the vapour pressure. So the case of pressure transient pulsations with cavitation in the downstream pipeline are analyzed to further verify the validity of the model using an impulse response function. Some experimental parameters of the downstream pipeline are listed in Table 6^29^. The test pipeline is composed by a copper pipeline and a transparent tube. The pressure transients are triggered by a steel ball of diameter 15 mm.

**Table 6:** Parameters of pressure transients with cavitation in the downstream pipeline.

Parameters	Values
Upstream tank pressure $p_{resu}$ (bar)	1.14
Pipe radius *r* (mm)	8
Pipe length *L* (m)	4.105
Water density $\rho$ ($\text {kg}/\text {m}^{\text {3}}$)	1000
Initial velocity $v_0$ (m/s)	0.65
Pressure wave velocity *a* (m/s)	1199.3

The number of impulse response of first order elements in Kagawa model is determined as ten for this case (*k* in Eq. (8) is equal to 10). For the model using an impulse response function, the series of simulation coefficients in the model using an impulse response function are calculated: $M=0.055$, $K=0.004$, and $k_1=1.98$. The all related parameters in different cases(without cavitation and with cavitation) are listed in Table 7. The best conclusion is found from Table 7 that the value of the parameter *K* is smaller than previously considered pipe length *L*=37.2 m and *L*=200 m, so *K* is proportional to the length of pipeline.

**Table 7:** Parameters from the model using an impulse response function in different cases.

Parameters	Without cavitation	With cavitation (upstream)	With cavitation (downstream)
Pipe length *L*(*m*)	37.2	200	4.105
$\varepsilon$	0.001	0.001	0.001
*M*	0.452	4.501	0.055
*K*	0.043	0.611	0.004

**Figure 9:**
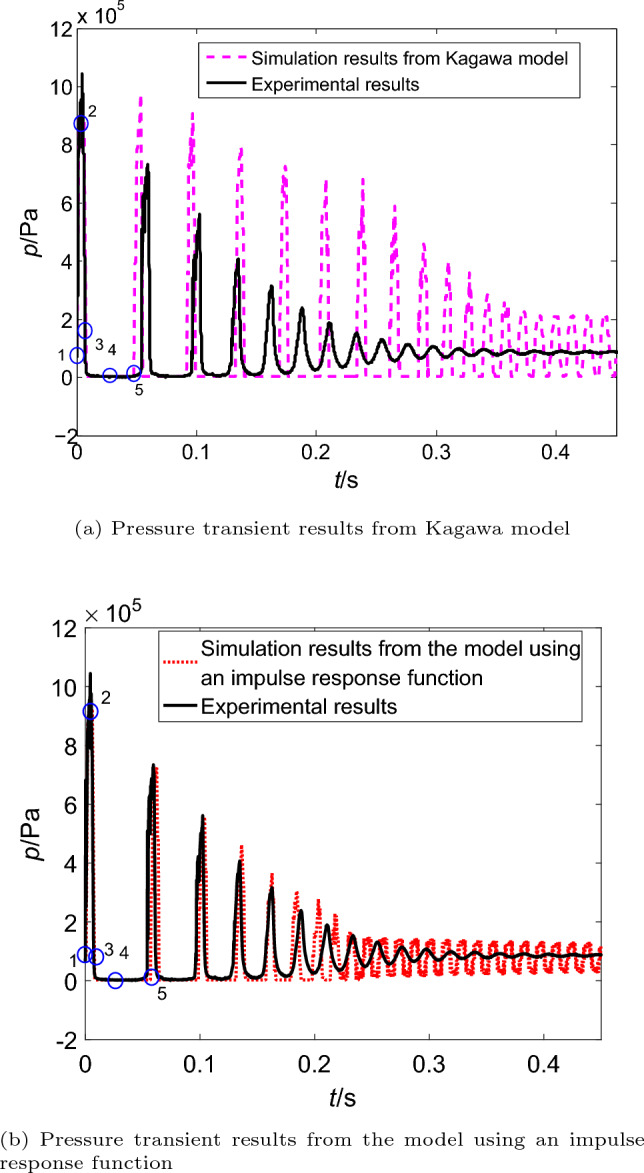
Comparison of simulation results and experimental data of pressure transients with downstream cavitation.

The solid line in Fig. 9 represents the experimental results. When the valve is rapidly closed, the pressure rapidly rises to its maximum value. Then it continuously drops to the vapour pressure as the pressure wave transfers to the upstream tank, and holds this transient value until approximately $t = 0.053$ s. The pressure still rises again then drops to the vapor pressure for about 0.035 s. For the third time, the pressure falls and stays at vapor pressure for about 0.026 s. At last, pressure pulsation gradually decays until it returns to the initial pressure $p_0$. The cavitation appears when the vapour pressure of the liquid is reached.

**Figure 10:**
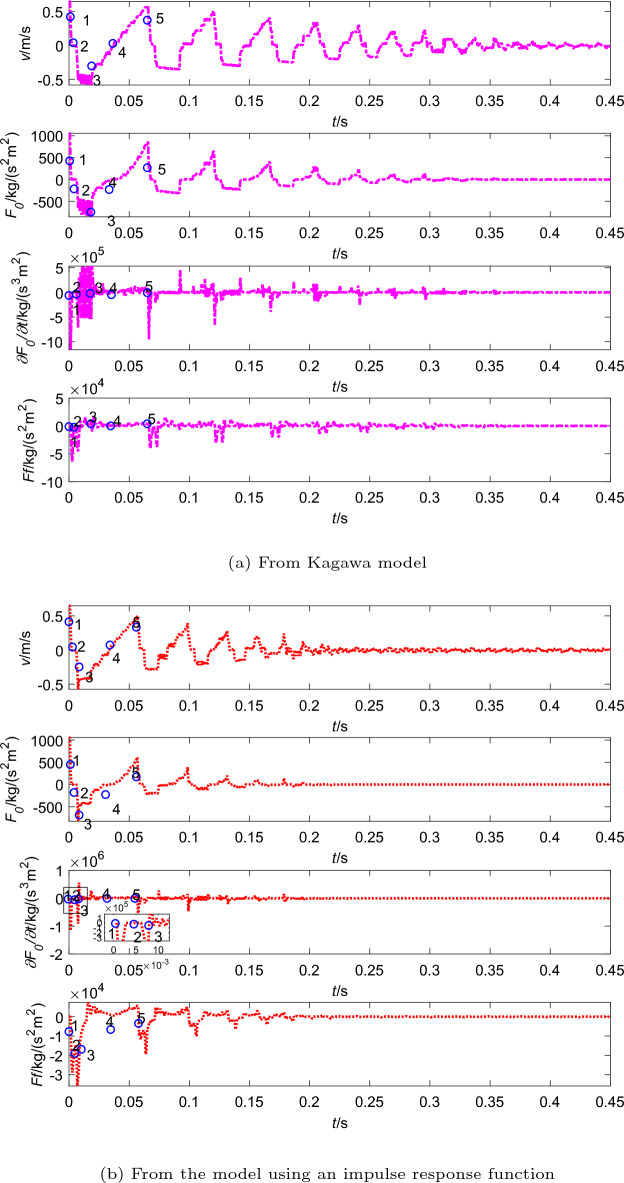
Steady and dynamic frictions with cavitation (in the pipeline with downstream valve).

And the pressure results obtained from Kagawa model and the model using an impulse response function are shown in Fig. 9a and b. The shock pressure is a positive value at the valve closing time, which makes the pressure goes up to $p+\Delta p$ when the liquid velocity drops to zero (see point 2 in Figs. 9 to 10^29^). And many differences exist between the two models. The Kagawa model’s simulation results (Fig. 9a) are consistent with the experimental data when the first wave peak appears, but the difference with the experimental results is evident from the second peak to the end. However, the simulation results of the model using an impulse response function are in good agreement with the experimental curve in Fig. 9b. It can be seen that the model using an impulse response function causes a very good damping of the pressure wave amplitude. Meanwhile, the steady friction and dynamic friction from two models are reported in Fig. 10a and b, respectively.

**Figure 11:**
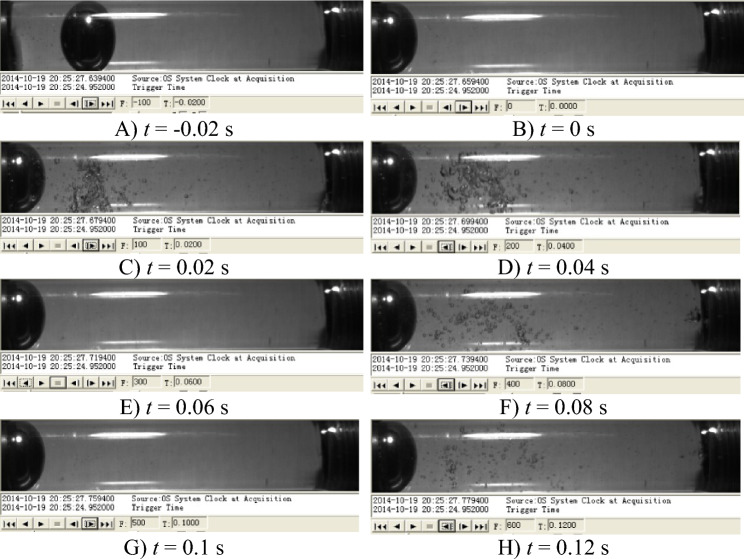
Growth and collapse sequence of cavities in the downstream pipeline^29^.

Figure 11 illustrates the growth and collapse sequence of cavitation in the tube, which are recorded by the high speed video camera^29^. The cavitation changes can be roughly estimated from the photograph in Fig. 11. Moreover, the trends of cavitation generate and collapse are also list in Table 8, which compared with the experimental data. When the pressure drops down to the vapor pressure, it emerges small expanded cavities(Fig. 11D), and the duration time of the first cavitation from the model using an impulse response function are closer to observed data (Table 8). Next, the drops down again to the vapor pressure. The second cavitation (Fig. 11F) occurs. At this moment, the cavities volume was smaller than the former cavities shown in Fig. 11. When the second cycle of pressure pulsations finishes, the cavities are observed again (Fig. 11H), and collapse at about 0.126 s. After about $t=0.2$ s, the pressure pulsation becomes an attenuated sinusoidal wave and the pressure is over the saturated vapor pressure all time, there is no cavitation (Fig. 9).

**Table 8:** Trends of cavitation generates and collapses in the downstream pipeline.

Times		Experimental results	Results from Kagawa model	Results from the model using an impulse response function
1st time	Start time (s)	0.008	0.008	0.008
	End time (s)	0.053	0.049	0.057
	Duration (s)	0.045	0.041	0.049
2nd time	Start time (s)	0.06	0.055	0.064
	End time (s)	0.092	0.121	0.099
	Duration (s)	0.035	0.037	0.035
3rd time	Start time (s)	0.100	0.098	0.105
	End time (s)	0.126	0.133	0.132
	Duration (s)	0.026	0.035	0.027

**Figure 12:**
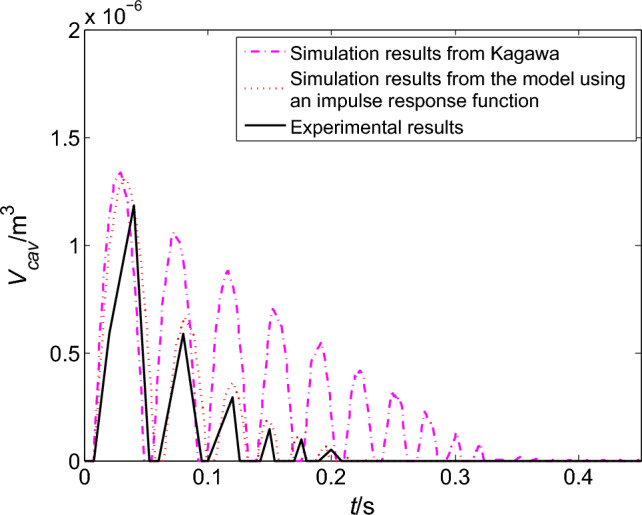
Comparisons of the cavitation volume simulation and experimental results in the downstream pipeline.

Combined with the information from Table 8 and the photograph in Fig. 11, an experimental curve of the change of cavitation volume over time can be obtained, which is showed as the solid line in Fig. 12. The change curves of cavitation volume with time can also be obtained from the different friction models, which are shown as dashed line and dotted line in Fig. 12. Compared with the simulation results, the cavitation simulated by the model using an impulse function is more consistent with experimental result. So it is concluded that the model using an impulse response function can not only predict the pressure transients with cavitation.

## Conclusions

In this paper, Kagawa model and the model using an impulse response function are compared for describing the dynamic friction force. And the simulation results of two models are given by comparing with the previous experimental data. Meanwhile, the steady friction ($F_0$) and dynamic friction ($F_f$) from two different models are analyzed. It is shown that the model using an impulse response function for transient pressure estimation has the following advantages:

1. The model using an impulse response function is closer to the experimental curves than Kagawa’s model for the three cases mentioned in this paper, which is reflected in terms of the phase differences and magnitudes of the pressure peaks.
2. Not only it accurately simulate the pressure transients without cavitation, but also availably predict the pressure transients with cavitation. And the changes in cavitation volume generated in low pressure pipelines can also be obtained.

However, the coefficient $k_1$ in the model of an impulse response function has not been determined by an exact way. So further studies will be focused on the parameter $k_1$, maybe it can be identified by using genetic algorithms, or other optimization methods. And it is necessary to testify the model using an impulse response function can be applied under other test conditions.

## Data Availability

The datasets used and/or analyzed during the current study are available from the corresponding author upon reasonable request.
